# Auditory Spatial Acuity Approximates the Resolving Power of Space-Specific Neurons

**DOI:** 10.1371/journal.pone.0000675

**Published:** 2007-08-01

**Authors:** Avinash D. S. Bala, Matthew W. Spitzer, Terry T. Takahashi

**Affiliations:** 1 Institute of Neuroscience, University of Oregon, Eugene, Oregon, United States of America; 2 School of Psychology, Psychiatry, and Psychological Medicine, Monash University, Victoria, Australia; Stanford University, United States of America

## Abstract

The relationship between neuronal acuity and behavioral performance was assessed in the barn owl (*Tyto alba*), a nocturnal raptor renowned for its ability to localize sounds and for the topographic representation of auditory space found in the midbrain. We measured discrimination of sound-source separation using a newly developed procedure involving the habituation and recovery of the pupillary dilation response. The smallest discriminable change of source location was found to be about two times finer in azimuth than in elevation. Recordings from neurons in its midbrain space map revealed that their spatial tuning, like the spatial discrimination behavior, was also better in azimuth than in elevation by a factor of about two. Because the PDR behavioral assay is mediated by the same circuitry whether discrimination is assessed in azimuth or in elevation, this difference in vertical and horizontal acuity is likely to reflect a true difference in sensory resolution, without additional confounding effects of differences in motor performance in the two dimensions. Our results, therefore, are consistent with the hypothesis that the acuity of the midbrain space map determines auditory spatial discrimination.

## Introduction

The ability to discriminate between stimuli is hypothesized to depend on the reliability of the change in activity of individual sensory neurons (e.g., [1]–[5]. Better discrimination is afforded by neurons whose firing rates change dramatically relative to the variability in the firing evoked by repetition of identical stimuli. For instance, a study of visual motion showed that individual monkeys with, on average, more sensitive and consistent neurons in the midtemporal cortical area (MT) were better able to discriminate changes in the direction of motion [6], strengthening the case for the involvement of area MT in motion discrimination.

An opportunity to further test the hypothesis is found in the auditory system of the barn owl (*Tyto alba*), a nocturnal bird-of-prey, renowned for its ability to localize sounds. The original survey of neurons of the auditory space-map in the external nucleus of the inferior colliculus (ICx; [7], [8]) demonstrated that the spatial receptive fields (SRFs) tended to be elongated vertically. The hypothesis-that better neuronal discrimination determines better behavioral performance-would predict that behavioral discrimination would be worse vertically than horizontally. Furthermore, if the space map was directly involved in auditory spatial discrimination, the difference in azimuth vs. elevation behavioral performance would be proportional to the difference in neuronal acuity of the space map neurons.

To test this hypothesis, we measured the minimum audible angle (MAA), which quantifies the abilities of owls to detect changes of sound source location in azimuth and elevation. The method of estimating the MAA is based on the pupillary dilation response (PDR). In the owl, the pupil dilates upon presentation of a sound and habituates with repetition of the same sound from the same location. The PDR recovers, however, if the sound source's location is perceptibly changed. The magnitude of the recovered PDR is proportional to the angular displacement of the source, making the PDR similar to a psychophysical rating task. Since pupillary dilation is mediated by the same motor circuits regardless of whether the source is displaced vertically or horizontally, differences in behavioral performance should only reflect differences in sensory resolution. Motor performance would be of concern, by contrast, in a gaze-directing task [9], where the motor circuitry and musculature would differ for vertical and horizontal movements.

Previously [1], we had found that the latency of the PDR was of the order of 19 ms, suggesting that the dilation response was too fast to be mediated by the forebrain, where response latencies of auditory neurons approximate 21 ms [10]. This fast response is probably mediated by the midbrain, where auditory neurons we sampled had a mean response time of 12.3 ms [11]. Thus, a promising location to examine the relationship between behavior and neuronal acuity is the ICx, where the space map is first constructed [12]–[15].

Below, we demonstrate that the vertical and horizontal MAAs differ by a ratio of about 2. We then show that azimuthal discrimination of ICx neurons is finer than elevation discrimination by a ratio of about 2, and that comparisons of the spatial resolution abilities of ICx neurons–assessed by incorporating magnitude as well as variance of firing rate changes in azimuth and elevation–yield a similar ratio. Just as studies based on lesions and microstimulation have implicated the ICx and its efferent target, the optic tectum, in acoustically-guided orienting behavior [16], the present study suggests its role in spatial discrimination.

## Results

### Behavioral acuity

**Figure 1:**
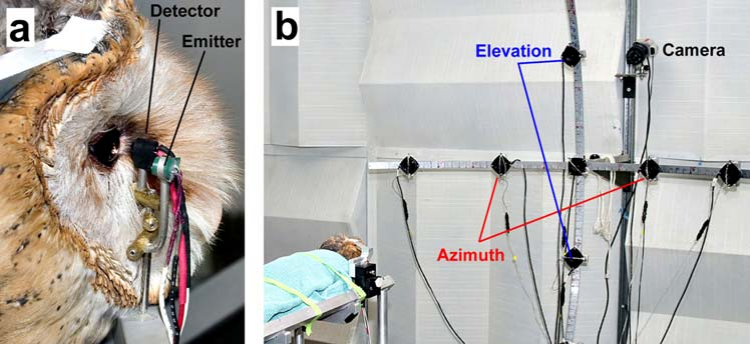
Measurement of spatial discrimination using the PDR. (A) A pupillometer, consisting of an IR detector and emitter (marked), is placed close to the cornea of the owl. The detector is placed about 6 mm from the eye, while the emitter is about 20 mm away. The owl is held immobile in a stereotaxic apparatus, allowing us to position the owl, repeatedly, in the same orientation *vis-à-vis* the pupillometer as well as the external sound sources. (B) Sound sources are placed on an array of two aluminum arms at right angles to each other, curved such that the center of curvature is a spot between the two ears of the owl. Speakers separated along the horizon of the owl were used to assess discrimination in azimuth (red), and speakers separated along the midline of the owl were used to assess discrimination in elevation (blue). The array is positioned such that the intersection of the arms is directly in front of the bird (azimuth 0°; elevation 0°). Each degree of angular displacement is marked on the arms of the array, and speakers can be moved to change angular separation. The subject is monitored using the IR camera, indicated here, during the experimental run. As far as possible, wiring is located so that it is behind the speaker array.

**Figure 2:**
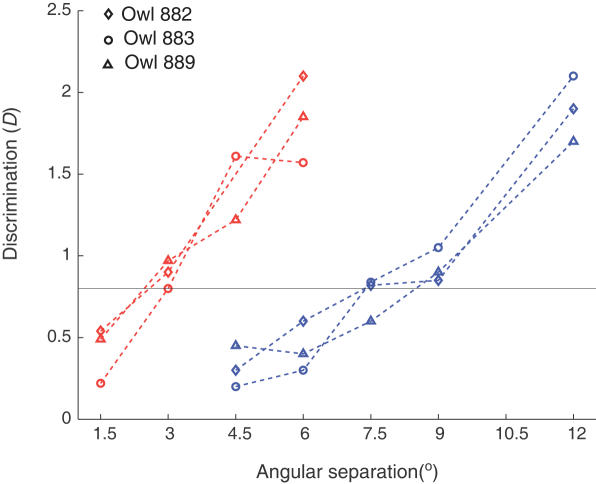
Behavioral discrimination, as measured by the PDR. Symbols and dotted lines represent discrimination values for each subject. By noting the points of intersection of each of the dashed lines with the discrimination functions, we can extract a ratio of elevation to azimuthal discrimination for a given value of *D*.

Spatial discrimination in azimuth and elevation was measured using habituation and recovery of the PDR in 3 owls. The magnitude of pupillary dilation evoked by a noise burst from the habituating location (Fig. 1) was compared to that of responses elicited by the same noise burst presented from a different location, and converted into the discrimination metric *standard separation (D)*, per Equation 1. The computed *D* was plotted against the distance between test and habituating loci (Δx) to generate psychometric functions (Fig. 2). The psychometric functions for elevation for individual subjects are well to the right of the functions for azimuth, indicating that vertical displacements are harder to discriminate than horizontal ones. Using an arbitrary discrimination threshold of *D* = 0.8, we observed that the azimuthal MAA was 3° in azimuth and 7.5° (2 birds) to 9° (1 bird) in elevation.

Thus the ratio between discrimination in elevation and in azimuth is about 2.5 (7.5/3), when the respective MAAs are compared. In addition, by interpolating between measured separations (Fig. 2; dashed red and blue lines), we could determine the angular separation at which a given value of *D* is attained in azimuth and in elevation. At all points along the interpolated discrimination curves, we observe discrimination ratios (elevation/azimuth) of 1.9 to 2.2.

### Neuronal space tuning

**Figure 3:**
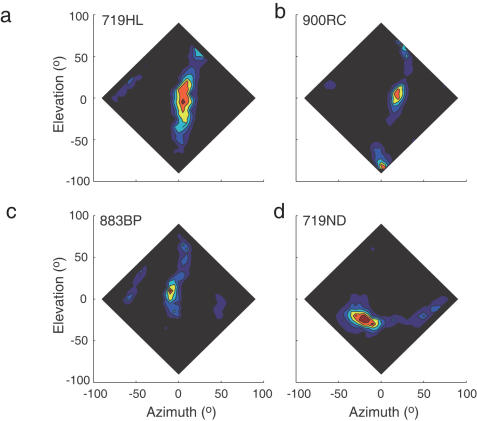
SRFs of four space-specific neurons. Black represents the spontaneous firing rate. Colors represent the firing rate, increasing from blue through to red. Note that for three of the neurons, the SRF is elongated in elevation. The neuron in (d) is atypical, in that the receptive field is more elongated in azimuth than it is in elevation. Such neurons were always tuned to low elevations.

We recorded from isolated space-specific neurons to determine whether the height/width ratios of neuronal tuning functions can explain the ratios of behavioral MAAs. Our conclusions are based on recordings from 62 neurons whose SRFs were characterized completely in virtual auditory space. Examples of recorded SRFs are shown in Fig. 3. The SRFs of most sampled units (54/62) were relatively elongated in elevation, in agreement with previous findings [7], [8]. A few neurons (3/62) with a flattened SRF were also observed, which were tuned to elevations more than 20° below eye level (e.g., Fig. 3d). In addition, several neurons were equally well tuned in azimuth and elevation (not shown).

**Figure 4:**
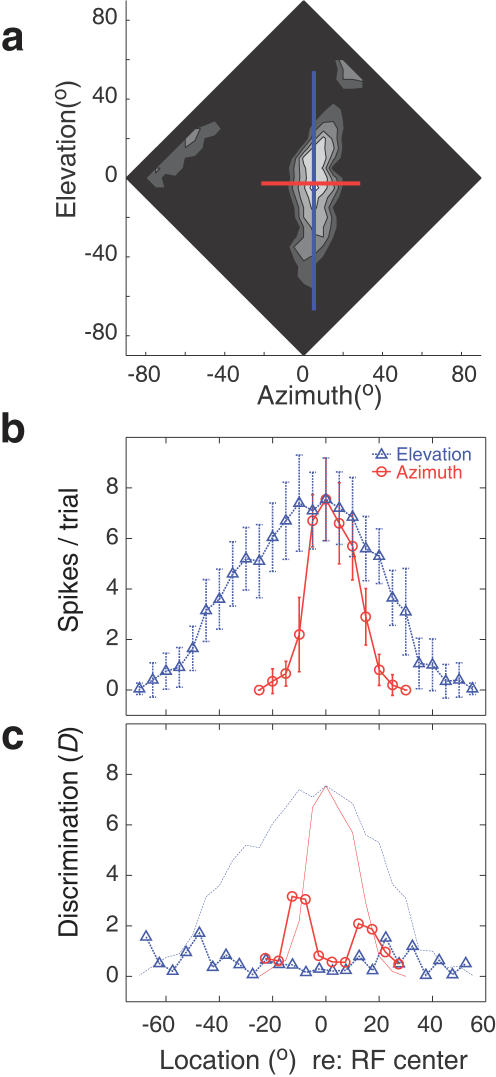
Azimuthal and elevation tuning and discrimination functions for a single space-specific neuron. (a) The SRF of the cell is shown; lighter shades correspond to higher response rates. The red and blue lines represent the locations used to estimate the azimuthal and elevational response functions, respectively. (b) Response profiles in azimuth (red) and elevation (blue) show that tuning in azimuth is finer than tuning in elevation. (c) Discrimination functions for a 5° separation were computed using data shown in (b), as per Eqn. 1. Response profiles for both azimuth and elevation are shown for reference. Note that maximal discrimination, especially as seen for azimuth, was achieved where rate of change of firing rate was maximal.

After charting the entire SRF in 5° increments, we examined the responses in 1° increments along vertical and horizontal transects through the neuron's best area (blue, red lines Fig. 4a). Sounds were presented 20 times from each location along the transect in order to calculate the mean and variance of the firing rate. Such data were plotted against angular separation to yield one-dimensional response profiles, which are shown in Fig. 4b. Note that the azimuthal response profile (red line and error bars) is much narrower than that in elevation (blue line and error bars). We used these neurophysiological data to assess neuronal acuity in several ways that allow us to compare neuronal and behavioral acuity directly.

### Neuronal spatial acuity

**Figure 5:**
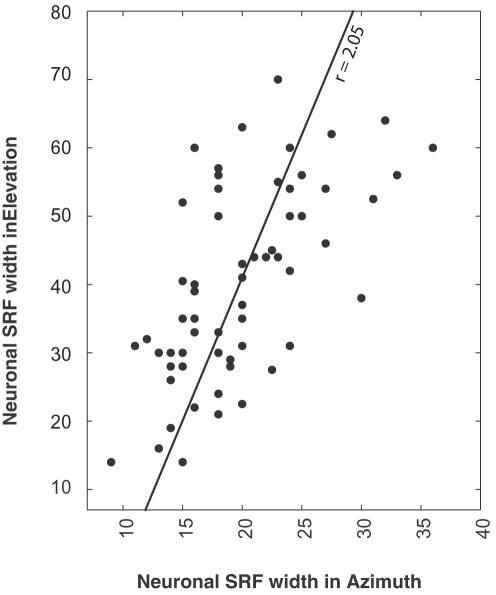
Azimuthal and elevation tuning in space specific neurons. The width at half the maximal firing response for each neuron in azimuth is plotted against its tuning in elevation. Neurons better tuned in elevation also tend to be better tuned in azimuth. The slope of the best fit line (dotted line) is 2.08, confirming that the width of elevation tuning is about twice that in azimuth.

A common way to estimate the spatial tuning of a neuron is to measure the width of its SRF at half maximal firing rate [7]. The average half-widths for tuning in azimuth and elevation for our sample of neurons were 19.9±5.7° in azimuth and 40.8±18.8° in elevation. Figure 5 shows the half-height breadth in elevation plotted against that in azimuth. The regression line shows that the height/width ratio is about 2.05, in good agreement with behavior.

Although the close agreement between the height/width ratios of behavioral acuity and neuronal acuity suggests a relationship between neuronal and behavioral sensitivity, it could be argued that such agreement is coincidental, since tuning curve half-width is an arbitrary measure, and its value depends on the chosen response rate criterion. Furthermore, analysis based on half-widths does not allow a direct comparison with the psychometric function. We therefore applied signal detection theory, which considers not only the average response rates of the neurons, but also their variance [2], [4], [17], [18].

A change of stimulus position can occur anywhere in space. A given neuron's contribution to the detection of that change across any arbitrary region can be estimated by computing the average of standard separations (*D_neuron_*) values across its SRF. The *D_neuron_*, calculated from neuronal responses to pairs of locations separated by 5°, is plotted against the center point of separation of each pair of locations in Fig. 4c (heavy lines w/symbols). Red circles represent the discrimination values for azimuthal separation, while blue triangles represent those for elevation. The azimuthal and elevation tuning functions are shown in light color for reference. Not surprisingly, discrimination was maximal where the change of firing rate is maximal, i.e., where the slopes of the response function were steepest. Note also that discrimination was higher towards the left of the peak in azimuth, where the slope of the tuning function was steeper for this neuron (Fig. 4b). Closer to the peak of the tuning function and to the base on either side, firing rates changed much more slowly, and the discrimination values were correspondingly lower.

It is also clear in Fig. 4c that discrimination performance in elevation was worse than that in azimuth. Here too, discrimination values were higher where the change in firing rate was maximal. However, at either foot, there was one location where discriminability was high, because variance at one location was very low. Such outlying spikes in discriminability were, however, not a consistent pattern. Inclusion or exclusion of these data produced no statistically significant change in the results, and were included in all our analyses.

**Figure 6:**
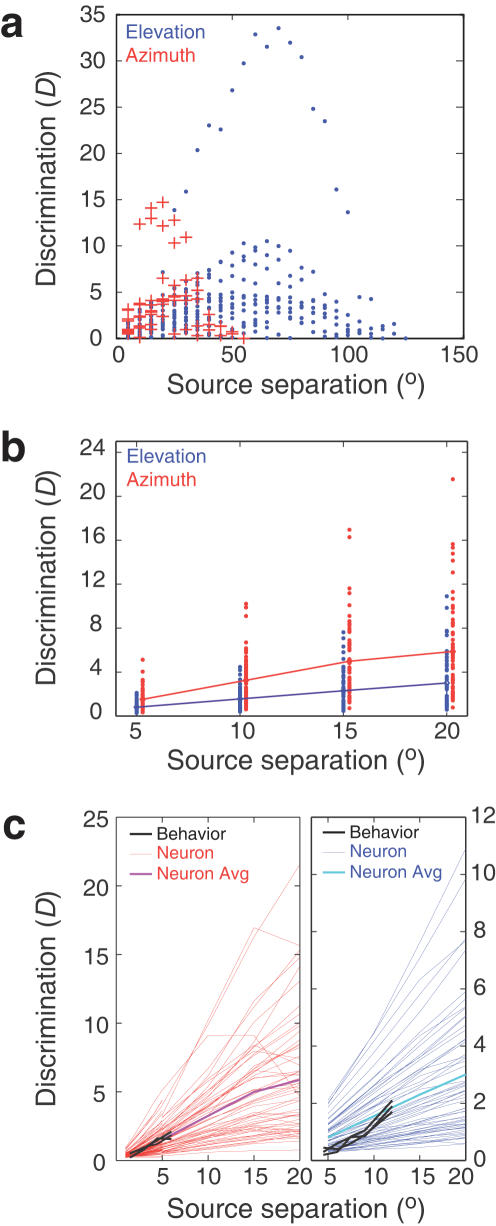
Azimuth discrimination by space specific neurons is better than elevation discrimination. (a) Individual discrimination values plotted here are from unit 719HL. Note that at lower spatial separations, azimuthal discrimination exceeds elevation, but maximal discrimination values are higher in elevation, albeit they occur at much larger separations. (b) Discrimination values plotted against angular separation. Elevation data are slightly offset to the right for clarity. Lines represent the mean discrimination computed from all neuronal pairs, such as those represented by symbols in (a). Note that azimuthal discrimination is roughly double that for elevation. (c) Comparison between behavioral data from individual birds (black) and neuronal discrimination. Data for azimuthal neuronal discrimination for separations <5° are drawn from a previous study [18]. Neuronal data shown by dots in (b) is shown as fine lines, in red for azimuth and in blue for elevation. Heavy line is the mean discrimination across all neurons. (Data for azimuthal neuronal discrimination for separations <5° are drawn from a previous study [18].) Note that neuronal discrimination falls somewhere close to the average neuronal discrimination. Neuronal data are pooled from our entire neuronal population.

In Fig. 6a, all of the *D_neuron_* values are plotted against the separations (Δx) for which *D_neuron_* values were computed for the neuron shown in Fig. 4 (unit 719HL). Thus, each symbol in Fig. 4c is represented by a symbol in Fig. 6a at 5° along the abscissa (red pluses: azimuth; blue dots: elevation). As angular separation increases, discrimination increases, because of a greater change in firing rate. However, for each separation, there will be many source pairs where discrimination will continue to be low, such as those that symmetrically straddle the peak. As source separation increases to more than half the width of the tuning function, discrimination values begin to fall, at angular separations far larger than the MAA. This fall in neuronal discrimination is expected, because locations at the foot of the tuning function on one side will now be compared to locations beyond the peak and the slope on the other side, and the available differences in firing rates will continue to decline. At smaller angular separations, where all available spatial separations are confined to one half of the receptive field, azimuthal discrimination exceeds that in elevation. However, when separations become very large, elevation discrimination continues to increase due to the larger distance between the foot and peak of the tuning function, while azimuthal discrimination has already fallen back to low values.

If we average the individual neurons' average *D_neuron_* values, we can estimate the spatial discrimination afforded by our entire sample of neurons. Figure 6b plots these averaged *D_neuron_* values against Δx. Each dot represents an individual neuron's average *D_neuron_*, for each spatial separation, and the lines represent means of the entire population of *D* values (The dots for azimuth and elevation are slightly offset horizontally for visibility). Note that azimuthal discrimination, represented by red dots, is significantly higher at all spatial separations than elevation discrimination (black dots). The two populations are significantly different at spatial separations of up to 20° (*t-test*, p<0.005).

Figure 6b, which depicts *neurometric* functions, shows the reliability with which our sample of neurons can signal changes in source position. Unlike the plots of tuning curve widths, the neurometric is directly comparable to the psychometric function shown earlier (Fig. 2). The psychometric functions from Fig. 2 are superimposed on the neurometric functions in Fig. 6c, and it is clear that the match is close for both azimuth and elevation. Not surprisingly, the ratio between vertical and horizontal discrimination is about 2 for both neurons and behavior at each of the Δx values tested, supporting the hypothesis that behavioral discrimination performance is dependent on neuronal reliability in the space map.

### Performance of a habituation-based model

To mechanistically link the neuronal responses to our behavioral paradigm, we had earlier developed a habituation-based model that replicated the observed behavioral and neuronal discrimination performances in azimuth [18]. Here, we evaluate the performance of this model in predicting spatial discrimination in azimuth and elevation.

**Figure 7:**
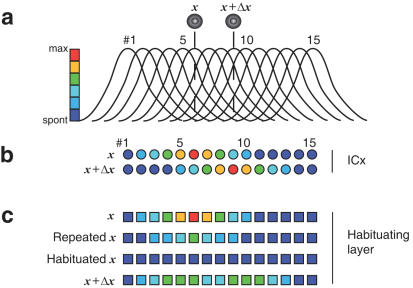
A habituation-based neuronal discrimination model. The illustration shows responses of SSNs and habituating neurons to sound source displacement along the azimuth. Space map neurons (colored circles) are represented by a series of tuning functions (a) centered over each cell (b). The space map neurons, which do not habituate, are assumed to project topographically to a layer of habituating neurons (c). The summed output of the habituating layer is assumed to be proportional to the pupillary response (not shown). The sound is initially presented from location *x* and repeated until each cell of the habituating layer habituates to the number of spikes received from its input space-map neuron. Thus an input firing rate that is identical to that transmitted earlier results in no output from the habituating layer, and the PDR is inactive. However, if the stimulus is presented from another location, *x*+Δ*x*, the focus of activity on the map moves, i.e., the firing rate of the space map neurons changes in neurons which encode the two locations. This change recovers the habituated neurons, and the pupil dilates. The degree to which each cell in the habituating layer recovers is proportional to the absolute value of difference between the input evoked at the new location (*x*+Δ*x*) and that evoked from the habituating location (*x*).

In this model, neurons of the space map, which do not habituate, were assumed to project topographically to a layer of habituating neurons, the summed activity of which was assumed to control the state of dilation of the pupil (Fig. 7; a–c). The first presentation of a stimulus evokes a focus of activity on the habituating layer activating pupillary dilation (Fig. 7a; upper layer in Fig. 7b). However, if the stimulus is repeated, habituation occurs [19], and this habituation is restricted to each neuron ([20]; Fig. 7c). We assumed that the decrease in firing rate was dependent on the initial firing rate of the SSN, as well as the variance of this input [21]. Thus, neurons that receive higher initial firing rates from the SSN layer are habituated to a greater extent. Also, habituating-layer neurons that receive inputs from space-map neurons with the lowest variance show greatest habituation. Conversely, neurons whose input neurons vary greatly in their responses from trial to trial, cannot effectively habituate because each change in firing rate acts as a ‘dishabituating' stimulus [22]. Practically, this can be achieved by including a computational step where the output from the space map is attenuated by a divisive process, and the magnitude of attenuation is proportional to the variance. Given this architecture, if the position of the source is changed, the neurons in the habituating layer receive a firing rate different from the rate to which they had habituated, re-activating their ability to respond (Fig. 7c).

**Figure 8:**
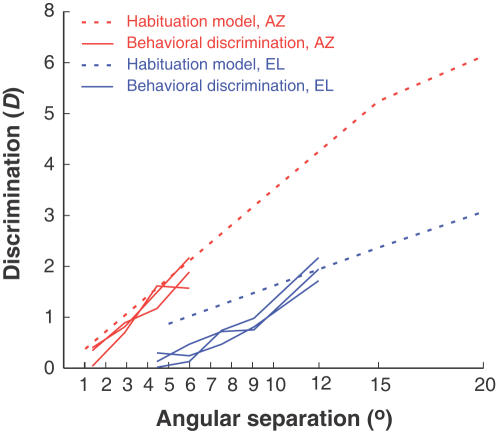
Comparison between neuronal and behavioral discrimination. The slope of the azimuthal discrimination function is about twice that of the elevation function in behavior (solid lines), as well as in the output of the habituation model (dashed lines). Note here that behavioral discrimination, shown by solid lines, is closely matched by the output of this computational model.

When the sampled neuronal responses are used as inputs, the model yields discrimination results that closely approximate behavior (Fig. 8), as well as the azimuth to elevation ratio that matches that seen in behavior. At behaviorally relevant angular separations–less than 15°–the elevation/azimuth discrimination ratio seen using this computational method is 2.

## Discussion

We demonstrated above that in the barn owl, spatial discrimination in azimuth exceeds that in elevation by a factor of about two. Because the PDR circuitry–which controls behavioral output–is the same whether the discrimination is made along the vertical or horizontal axes, this difference in acuity is likely to reflect the reliability of only the sensory apparatus and not a combination of the reliability in the motor and sensory segments. Indeed, we found that the RFs of neurons in the auditory space map are about twice as tall as they are wide. This ratio of neuronal acuity in elevation and azimuth was also observed with discrimination metrics derived from signal detection theory, and analysis with a computational habituation model (Table I). Studies based on lesions [16], [23] as well as microstimulation [24] have implicated the space map in auditory orientation. The results of the present study suggest that azimuth and elevation acuity of the space map are faithfully reflected in spatial discrimination of the barn owl, suggesting an additional involvement of the space map in auditory spatial discrimination.

**Table 1:** Behavioral and neuronal discrimination ratios.

Discrimination	Azimuth(°)	Elevation(°)	Ratio (El./Az.)
Behavioral discrimination (*D* = 1.0)	3.5	8.4	2.4
Behavioral discrimination (*D* = 1.8)	6.0	12.0	2.0
RF tuning widths	20.0	41.0	2.1
Habituation model (*D* = 2.0)	5.7	12.4	2.4

Behavioral discrimination was assessed at a resolution of 1.5°, and neuronal discrimination was assessed at a resolution of 5°. Neuronal ratios values shown below were obtained by noting the angular separations which corresponded to arbitrarily selected discrimination values.

### Behavioral acuity

Our measures of auditory spatial discrimination revealed a clear difference between performance in azimuth and in elevation. Spatial hearing in the barn owl has previously been analyzed by evaluating the owl's ability to orient toward the source. Konishi [25] first measured spatial acuity by the pattern of talon marks made by an owl striking a concealed sound source and reported that acuity in elevation (7°) was not significantly different from that in azimuth (5°). Knudsen et al. [9] measured localization in a head-pointing task and showed that when the target was within the frontal 10° of azimuth or elevation, errors in azimuth and elevation were indistinguishable from each other. At eccentricities greater than 50° elevational localization accuracy was significantly worse than azimuthal accuracy. However, Knudsen and colleagues also point out that the owls were reluctant to localize sounds at extreme elevations which may have contributed to a loss of accuracy in elevation. More recently, Poganiatz and colleagues [26], [27], using a head pointing task in VAS, demonstrated that while localization of eccentric sources in elevation is more error-prone than in azimuth, localization accuracy of centrally located sources was roughly equal in azimuth and elevation. The results from this VAS study are confounded by the fact that the birds were not explicitly trained to aim their heads at the target but only to make head turns toward the half of the frontal hemisphere (left, right, upper, lower) in which the targets were located.

Thus, our study of spatial *discrimination* showed a robust difference between vertical and horizontal acuity, whereas those based on spatial *orientation* have produced more equivocal results. While the methodological differences cannot be ignored, this difference may also reflect a difference in the way that the space map is used for the two tasks.

### Neuronal space tuning

That SSNs in the barn owl midbrain are better tuned in azimuth than in elevation was first reported by Knudsen and Konishi [7], [8]. While all subsequent neurophysiological examinations have confirmed this result, the impact of such a discrepancy on behavior has not been discussed or described. The present results show that the difference in horizontal and vertical acuity is consistent with the aspect ratio of the RFs of the SSNs.

The shape of spatial RFs is related to the distribution of the binaural cues across space and the sharpness and variance of neuronal tuning to the binaural cues. In the barn owl, interaural differences in timing and level (ITD and ILD), which are subserved by anatomically parallel and physiologically independent pathways, serve as cues for localization [13], [28]–[31]. Measurements of the filtering properties of the barn owl's ears [32] show that ITD varies as a function of azimuth, reaching maximal values of about 200 μs, and remains relatively constant across frequencies. ILD, like ITD, varies with azimuth at low frequencies, but at frequencies above 4 kHz, the axis along which ILD varies becomes increasingly vertical and non-monotonic

One possible reason for the vertical elongation might be that the cross-correlation-like process, with which ITD is thought to be computed [33], provides for sharper tuning curves and less error than the inhibitory, subtractive process thought to be involved in the computation of ILD [29], [34], [35]. On the other hand, if we assume that neuronal jitter is comparable in the ITD and ILD pathways, receptive-field shapes would be determined by the steepness of the ITD and ILD gradients across space [14], [15], [36]. The latter predicts that the aspect ratio of RFs as well as that of perceptual acuity would depend in a predictable fashion on the frequency and location at which acuity is estimated.

### Neuronal Codes and Perceptual Acuity

The discrepancy in the accuracy with which owls could aim their heads at an auditory target and the half-height width of their spatial RFs had led to assertions that owls gathered information from numerous coarsely-tuned neurons to achieve the high behavioral accuracy [37], [38]. More recent studies of the IC of guinea pigs and gerbils showed that such coarse coding processes need not be invoked [3], [4]. The change of firing rates evoked in the IC by a change in ITD was reliable enough to afford resolution in ITD discrimination observed in humans, if the guinea pig or gerbil neurons used a “lower-envelope” strategy [39] wherein the best neurons are relied upon. McAlpine and colleagues added that small headed mammals may depend on the slope of ITD functions instead of the peaks to localize sound. This idea also explained why the peaks of ITD tuning functions were often at ITD values beyond the physiological range of these mammals–putting the peaks at these large ITDs places the slope at the midline, where perceptual acuity is assumed to be highest [40], [41].

The apparent dichotomy between small-headed mammals, which are thought to use a slope code, and owls, which are argued to use a peak-based, “local code” (i.e., a space map) for sound localization, was recently postulated by [42]. However, this may be more a dichotomy in the tasks used to measure sound localization. If the task is one of discrimination, where the subject must report whether (or by how much) two sounds differ in their location, then it is reasonable to hypothesize the involvement of a slope code, and to predict that slopes should fall where behavioral acuity is highest. If, however, the task is one of orientation, where subjects must point their heads (or eyes) at the source, it seems simplest to invoke a space map where the focus of neural activity can be used to direct a saccade [43]. The owl's space map can function, not just in the latter mode as an auditory display, but also as a slope code. Because the cells of the space map have sharp RFs across all of frontal space, there will inevitably be neurons that dramatically change their firing rates when a source is moved from one location to another in a discrimination task. These are the cells along skirts of a neural image, and they form the basis of a slope code.

Our analysis postulates that the ability of the owl to discriminate changes in spatial location is dependent on the variance that has accumulated in the sensory pathways up to the ICx. However, we do not have an independent estimate of the contributions of motor error on the behavioral response. A recent analysis of tracking of visual targets in primates [44] suggests that in the visual system at least, errors in sensory estimates of stimulus parameters are the main contributors to behavioral output, and that contributions from the motor system are less obvious. The fact that degradation in neuronal sensitivity in the owl (elevation vs. azimuth) causes a proportional degradation in behavior suggests that the variances of the motor system do not influence behavioral predictions of the habituation-based model.

## Materials and Methods

### Behavior

Spatial discrimination behavior was measured using a discrimination assay based on the habituation of the acoustically evoked pupillary dilation response, details of which are available elsewhere [1]. The PDR was measured with an infrared pupillometer whose output voltage was plotted as a function of time, aligned with the onset of the stimulus. The magnitude of a PDR was defined as the area under this curve over the 2 s interval following stimulus onset. We compared the magnitudes of the PDR elicited by habituating and test stimuli using the discrimination metric *standard separation* (*D*) [45]:
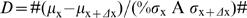
(1)The quantities μ_x_ and μ_x+Δx_ refer to the mean magnitudes of PDR evoked by habituating and test stimuli respectively. Similarly, σ_x_ and σ_x+Δx_ refer to the standard deviation of the PDR elicited by habituating stimuli and test stimuli. The index standard separation does not require that the distributions be normal or that the variances be equal [45].

Stimuli consisted of reproducible noise bursts with flat spectra (within 1 dB) between 3 kHz and 11 kHz, presented from speakers arrayed as shown in Fig. 1b at a distance of 1.98 m from the bird. All stimuli were presented at 52 dB SPA. The owl's ability to discriminate between two sound sources, separated in either azimuth or elevation, was investigated in 1.5° increments. Azimuthal discrimination was assessed using sound sources that straddled the midline at the bird's eye-level. Elevation discrimination was assessed using sound sources that straddled eye-level, and were placed at the midline of the subject. Below, negative azimuths and elevations refer to loci to the left of midline and below eye level, respectively.

Sound sources were aligned as closely as possible such that a source placed at 0° azimuth and 0° elevation would cast an image onto the retina at the visual fovea, or *area centralis,* of the barn owl's eyes [46]. This alignment was established at the time the headplate was mounted to the skull, ensuring that sound sources could be placed stereotaxically at reliable and reproducible locations relative to the barn owl's eyes and skull.

### Neurophysiology

Neuronal acuity was assessed in three barn owls under nitrous oxide anesthesia. The RF of isolated space-specific neurons was determined by presenting sounds from the frontal hemisphere, using a virtual auditory space paradigm based on individualized HRTFs [32]. Neurophysiological recordings were obtained from 241 isolated units in the inferior colliculus of the barn owl. Their location and response characteristics suggest that the vast majority of these were located in the external nucleus of the inferior colliculus (ICx). Detailed analysis was confined to clearly isolated units which had one dominant peak of sound-evoked activity in their SRFs, and in which secondary peaks, if any, were largely suppressed. Some units used in the present study have also been included in previous studies by [11], [18].

To compute the standard separation for neural responses, *D_neuron_*, we adapted Equation 1 such that μ_x_ and μ_x+Δx_ represented the mean firing rates between two adjacent loci separated by Δx and σ_x _and σ_Δx_ the respective standard deviances.

### Modeling

The habituation model attempts to reproduce the essential features of our behavioral paradigm, the habituation and recovery of the PDR [11], [18]. Neurons in the ICx–which do not habituate to repeated presentation of the stimulus (Fig. 7b)–project topographically to a layer of habituating neurons, where the responses to sound (HS) decline upon repeated presentation (Fig. 7c). Since spike rate is the only manifestation of stimulus identity that is transmitted from the ICx to this habituating layer, it is implied that the cause of habituation is the repeated exposure of a habituating cell to the same number of incoming spikes. The magnitude of habituation within each neuron is proportional to its incoming spike rate, such that all neurons that previously responded to the HS cease to respond (Fig 7c; third row). When sound is presented from a different location (test stimulus: TS), the firing rate of many space map neurons that also responded to the HS is changed. Some SSNs that responded to the HS cease to fire, while a new set of SSNs that did not respond to the HS will now fire in response to the TS. Each of these changes is transmitted as a change in output firing rate from each ICx neuron to its corresponding habituating layer neuron. The HL neurons, which were habituated, had ceased to respond to a *particular incoming firing rate*. Thus, the change in incoming firing rate from the ICx neuron will cause the HL neurons to recover (Fig 7c; fourth row). The recovery is proportional to the change in firing rate: neurons that experience the largest changes in firing rate will also recover most, and fire at the highest rates.

The model also incorporates the trial-to-trial variance in firing of the space-specific neurons which diminishes the ability of HL neurons to respond to changes in firing rate induced by a test sound. This variance was included by scaling the output of each ICx neuron by its standard deviation. Thus, space-specific neurons with small variance in their response will contribute more to the HL neurons' response, while those with large variances in their response will have a proportionately reduced contribution to the output of the HL neurons. This property of our hypothetical HL neurons is reminiscent of neurons in the fly's visual system whose firing rates scale with the variance in prior stimulus inputs [21].

Each of the two components, mean and variance, is included in the computation as follows. First, the output of each ICx neuron, R, is normalized by the variance of response to the habituating stimulus (V_HS_; Eqn. 2):
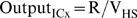
(2)The output of each space map neuron was normalized by passage through a transformation function, causing the spike rate to be scaled down. Note that when responses are normalized to the standard deviation (V_HS_) of R_HS_, then Output_ICx_ equals the z-score. The normalized output is then sent to the HL neurons, where the responses to sound from the HS (R_HS_) decline upon repeated presentation. This results in a habituation gradient, where the output of the habituating cell declines to presentation of sounds from the habituating location, but declines less as the test source moves further from the habituating location.
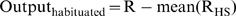
(3)While responses to the habituating stimulus decline, presentation of stimulus from a new location causes the neurons to discharge (Eqn. 3). Thus, habituating layer neurons fail to respond to stimulus presented from the habituating location (Fig. 7c; third row), but respond to presentation of stimulus from a new location (Fig. 7c; fourth row).

The output of each HL neuron is summed and averaged [47]

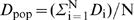
(4)The quantity *D*_pop_ describes the population activity in the habituating layer, which is transferred to the owl's pupil. The output of this model (Fig. 8; dotted lines) yields a close match with behavior (Fig. 8; solid lines). Note that the pooled output of the population is scaled here by the total number of participating neurons N; it is conceivable that the scaling factor could be different.
